# Stronger zonal convective clustering associated with a wider tropical rain belt

**DOI:** 10.1038/s41467-019-12167-9

**Published:** 2019-09-19

**Authors:** Max Popp, Sandrine Bony

**Affiliations:** Laboratoire de Météorologie Dynamique (LMD/IPSL), Sorbonne Université, Centre National de la Recherche Scientifique (CNRS), École Polytechnique, École Normale Supérieure, 4 Place Jussieu, 75005 Paris, France

**Keywords:** Atmospheric science, Atmospheric dynamics, Climate change, Climate and Earth system modelling

## Abstract

Deep convection can exhibit a large diversity of spatial organizations along the equator. The form of organization may affect the tropical large-scale motions of the atmosphere, but observational evidence is currently missing. Here we show using observations that when convection along the equator is more clustered in the zonal direction, the tropical rain belt widens in the meridional direction, and exhibits a double-peak structure. About half of the influence of the convective clustering on the width of the rain belt is associated with the annual cycle and the other half is associated with unforced climate variability. Idealized climate model experiments show that the zonal convective clustering alone can explain the observed behavior and that the behavior can be explained with an energetic framework. This demonstrates that the representation of equatorial convective clustering is important for modeling the tropical rainfall distribution accurately.

## Abstract (web summary)

How the spatial patterns of deep convection affect the large-scale dynamics of the atmosphere remains an open question. Here, it is shown that if convection along the equator is clustered, the tropical rain belt widens and exhibits a double peak structure.

## Introduction

A major open question in climate science is whether the spatial and temporal organization of deep convection (known as convective clustering) in the tropics matters for the climate and the climate response to natural or anthropogenic perturbations^1^. The convective clustering has been shown to have a large influence on the atmospheric state in idealized simulations of radiative-convective equilibrium^2–5^. In general, more clustered states are associated with larger subsiding regions, a drier atmosphere, higher tropospheric temperatures and a larger variance of water vapor^6^. In Earth’s tropics, deep convection predominantly occurs in the intertropical convergence zone (ITCZ) sometimes also referred to as rain belt^1^, which naturally raises the question if the organization of deep convection within the ITCZ also affects the tropical climate in a similar way. The ITCZ and its properties have been identified in different ways in the past by considering the regions of strong precipitation, strong low-level meridional mass convergence, strong tropospheric ascending motions or regions with changes in the sign of the mean-meridional energy flux^7–12^. Using these different methods, it has been shown that the position and the width of the ITCZ are affected by changes in the atmospheric energy budget^8–20^. Since the convective clustering has been shown to affect the atmospheric energy budget^6,21^, we suspect a link between the convective clustering and ITCZ properties.

On Earth, the organization and meridional position of intense convection can vary zonally due to zonal variations in the boundary conditions such as the presence of continents, orography and areas of high and low sea-surface temperatures^22–27^. Furthermore, atmospheric waves and extratropical disturbances can induce zonal variations in convective activity^27–31^. These zonal variations can occur on a spatial scale ranging from a few kilometers^32,33^ to planetary scale patterns as in the case of the Madden Julian Oscillation (MJO)^34–36^. Here we investigate how different forms of zonal convective clustering affect the ITCZ and the tropical large-scale circulation.

Using observations we show that when convection is clustered along the equator the ITCZ widens and exhibits a double-peak structure. We can simulate the same behavior when imposing zonally varying patterns of evaporation in numerical experiments, implying that the zonal convective clustering can by itself cause the observed behavior. The results of our simulations suggest that this can be explained by the reduced atmospheric cloud-radiative effect in the equatorial atmosphere that reduces the net atmospheric energy uptake. This leads to a weakening of the meridional atmospheric energy transport and the large-scale circulation, and thus to a widening of the ITCZ. About half of the variance explained between the zonal convective clustering and the width of the ITCZ is associated with the seasonal cycle, and the other half with unforced natural variability. The results highlight the importance of the convective clustering for modeling the tropical large-scale circulation and the precipitation distribution accurately.

## Results

### Characterizing zonal convective clustering and ITCZ properties

**Fig. 1:**
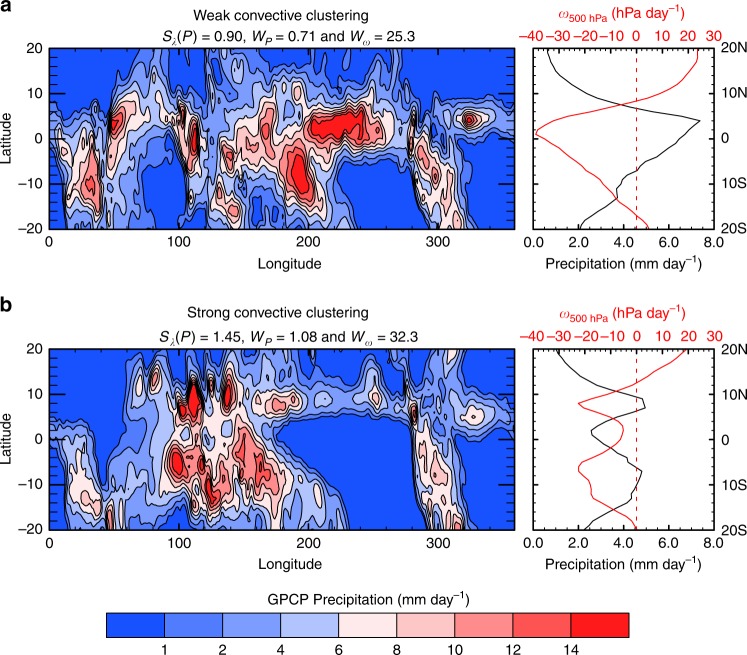
Examples of observed weak and strong convective clustering. **a** The mean precipitation of the December month with the weakest convective clustering and **b** the December month with the strongest convective clustering. In the case of strong convective clustering the zonal-mean precipitation distribution (shown on the right) is wider than in the case of weak clustering. The red line on the right-hand side panels shows the vertical pressure velocity at the 500 hPa level (${\omega }_{\text{500hPa}}$) inferred from the ERA-interim reanalysis dataset for the same months. Note that even in the strong clustering case with a distinct precipitation-inferred double-peak structure of the intertropical convergence zone (ITCZ) there is just one upwelling region (with negative ${\omega }_{500\text{hPa}}$), and thus the dynamical width inferred with this quantity comprises both peaks. ${S}_{\lambda }(P)$ denotes the zonal convective clustering, ${W}_{P}$ the precipitation-inferred and ${W}_{\omega }$ the dynamically inferred width of the ITCZ. Note that the precipitation displayed on the two map-plots was smoothed for better visibility using a nine-point smoothing algorithm

We start by investigating the relationship between the zonal clustering of convection and the observed meridional precipitation distribution using the Global Precipitation Climatology Project (GPCP) dataset^37,38^. We characterize the degree of zonal convective clustering by the spatial concentration of convection in the zonal direction along the equator, with strong (weak) clustering corresponding to a small (large) fractional area covered by convection. Several metrics can be used for this purpose (see Methods section). We will focus here on the monthly mean of the zonal standard deviation of the meridional average of precipitation from 6 S to 6 N, normalized by the mean precipitation over the equatorial band from 6 S to 6 N, a metric referred to as ${S}_{\lambda }(P)$. The idea behind this metric is that the zonal standard deviation of precipitation is larger when the precipitation, and thus the convection, is spatially concentrated, whereas it is small when precipitation is uniformly distributed. An example of situations with strong and weak clustering is shown in Fig. 1. The other metrics used for zonal convective clustering here, are, first, the subsidence fraction in the band from 6 S to 6 N and, second, the minimum area fraction that receives 80% of precipitation in the band from 6 S to 6 N. This way of characterizing convective clustering is reminiscent of previous work on convective clustering in radiative-convective equilibrium configurations where it was found that stronger convective clustering was associated with a larger spatial variance of precipitation and moisture^2–6,39,40^. Calculating the convective clustering in the region from 6 S to 6 N makes the interpretation of the results difficult in months in which the ITCZ moves far away from the equator, notably in boreal summer. Therefore, we expect any relation between the convective clustering and other variables to be most meaningful when the zonal-mean tropical precipitation distribution is approximately symmetric about the equator (which occurs between November and May). The width of the ITCZ is characterized by using the precipitation. More precisely we define the width of the ITCZ ${W}_{P}$ as the ratio between the mean precipitation from 15 S to 15 N and the mean precipitation from 6 S to 6 N. Thus, the larger the value of ${W}_{P}$ the wider the ITCZ. Note that this definition treats cases of ITCZs with single and double-peak structure identically, implying that for double-peak structure the width is tied to the width of the bulk low-latitude precipitation, rather than to the width of each individual peak. Additionally, we use a dynamical indicator for the width of the ITCZ (${W}_{\omega }$) in a few cases, namely the width of mean ascent at 500 hPa^15^ which has been a popular indicator in recent studies^10,12,15,18^. The two metrics for the width are positively correlated when all months are considered and highly correlated when only months with a hemispherically symmetric distribution of precipitation in the tropics are considered (Supplementary Table 1).

### Zonal clustering in observations and in simulations

**Fig. 2:**
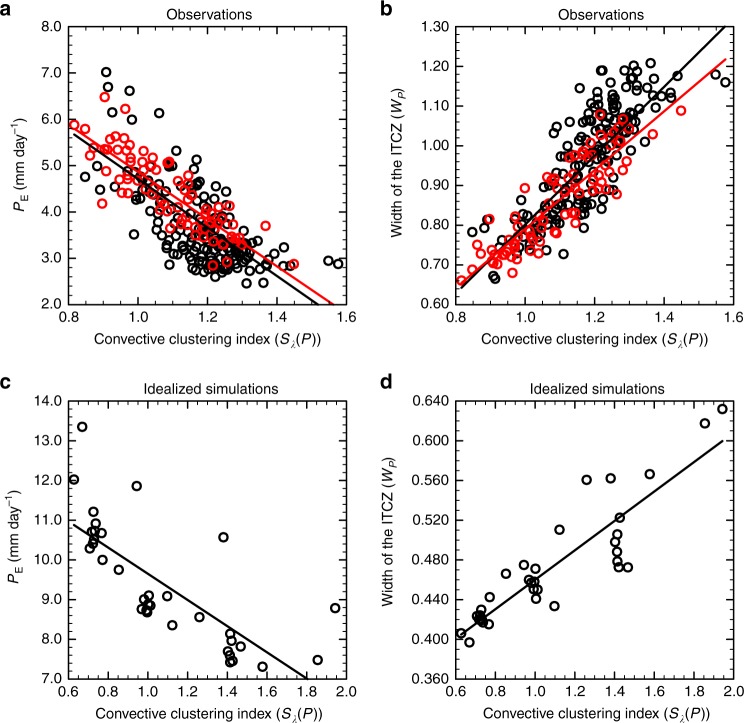
Properties of the intertropical convergence zone as a function of convective clustering. **a** The monthly means of the equatorial mean precipitation ${P}_{\text{E}}$^56^ as a function of the zonal convective clustering (*S*${}_{\lambda }(P)$) for the GPCP observational dataset from October 1996 until December 2016. **b** The same but for the precipitation-inferred width of the intertropical convergence zone (${W}_{P}$). Red circles denote the months with a tropical precipitation distribution that is symmetric about the equator and black circles those with an asymmetric distribution. The red line is obtained by linear regression to the red circles and the black line is obtained by linear regression to all (red and black) circles. **c** The same as **a** but for a suite of model simulations associated with different zonal convective clustering, with each marker representing the statistically steady-state mean value of a simulation. **d** The same as **c** but for ${W}_{P}$

The results suggest that stronger zonal convective clustering is associated with less equatorial precipitation and with a wider ITCZ (Fig. 2, Table 1). This is both true for the width inferred from precipitation ${W}_{P}$ and for the dynamically inferred width ${W}_{\omega }$. The behavior is particularly pronounced during months with a hemispherically symmetric tropical precipitation distribution (Fig. 2, see Methods section for our definition of hemispheric symmetry). Moreover, in months with a symmetric precipitation distribution stronger zonal convective clustering is also associated with a more pronounced double-peak structure. Note that in monthly zonal-mean ERA-interim reanalysis data^41^, double-peaked ITCZs are not associated with an equatorial anti-Hadley circulation as is sometimes the case in idealized simulations^42^. Thus even in cases with a double-peak ITCZ, there is no zonal-mean descending motion between the peaks, and the dynamical width of the ITCZ as defined in the methods therefore encompasses both (precipitation) peaks of the ITCZ. So, both from a dynamical and from a precipitation point of view, double-peaked ITCZs are associated with wider ITCZs in months with a symmetric precipitation distribution (Supplementary Table 1). A similar behavior has also been found in idealized aquaplanet simulations^11,16^.

**Table 1:** Relation between clustering and the tropical

	${P}_{\text{E}}$	${W}_{P}$	${W}_{\omega }$	${\phi }_{\text{S}}$	${T}_{\text{2m}}$	${\Delta }_{\lambda }{T}_{\text{2m}}$	${\Delta }_{\phi }{T}_{\text{2m}}$	${\nabla }_{\lambda }\cdot q{\bf{u}}$	${\nabla }_{\phi }\cdot q{\bf{v}}$	${H}_{\text{rad}}$	${H}_{\text{ACRE}}$
${S}_{\lambda }(P)$ (GPCP all)	(−) 58%	(+) 71%	(+) 39%	(+) 3%	(−) 62%	(+) 43%	(−) 1%	(+) 67%	(−) 61%	(−) 31%	(−) 66%
${S}_{\lambda }(P)$ (GPCP sym)	(−) 72%	(+) 78%	(+) 70%	(+) 56%	(−) 70%	(+) 32%	(+) 4%	(+) 74%	(−) 71%	(−) 53%	(−) 72%
${S}_{\lambda }(P)$ (simulations)	(−) 58%	(+) 82%	(+) 78%	(+) 14%	(−) 74%	(+) 55%	(−) 74%	(+) 82%	(−) 71%	(−) 65%	(−) 74%

The Table shows the variance explained (square of the correlation coefficient) between the zonal convective clustering (${S}_{\lambda }(P)$) and different climate variables as well as the sign of the correlation coefficient between these. The correlation coefficients are calculated using monthly mean data from observational (GPCP data for precipitation-inferred quantities and CERES-EBAF for energy fluxes) or from the ERA-interim reanalysis datasets (for all other quantities) and statistically steady-state mean data for the aquaplanet simulations (last row). ${P}_{\text{E}}$ denotes the equatorial mean precipitation, ${W}_{P}$ the precipitation-inferred and ${W}_{\omega }$ the dynamically inferred width of the intertropical convergence zone (ITCZ), ${\phi }_{\text{S}}$ denotes the distance between the two peaks in zonal-mean precipitation following ref. ^56^, ${T}_{\text{2m}}$ denotes the average 2m temperature from 6S to 6N, ${\Delta }_{\lambda }{T}_{\text{2m}}$ the zonal standard deviation in 2m temperature, ${\Delta }_{\phi }{T}_{\text{2m}}$ the difference in average 2m temperature between the zonal band from 6 S to 6 N and the two zonal bands from 10 to 16 degrees latitude, ${\nabla }_{\lambda }\cdot q{\bf{u}}$ the zonal standard deviation of zonal moisture convergence at the equator normalized by the equatorial precipitation and ${\nabla }_{\phi }\cdot q{\bf{v}}$ the mean meridional moisture convergence. ${H}_{\text{rad}}$ denotes the vertically integrated atmospheric radiative cooling between 6 S and 6 N calculated from the CERES-EBAF data set and ${H}_{\text{ACRE}}$ its cloud-radiative contribution (only from March 2000 to December 2016). Values in the first row were calculated taking all months into account, whereas values in the second row only took the values for the months with a symmetric precipitation distribution about the equator

**Fig. 3:**
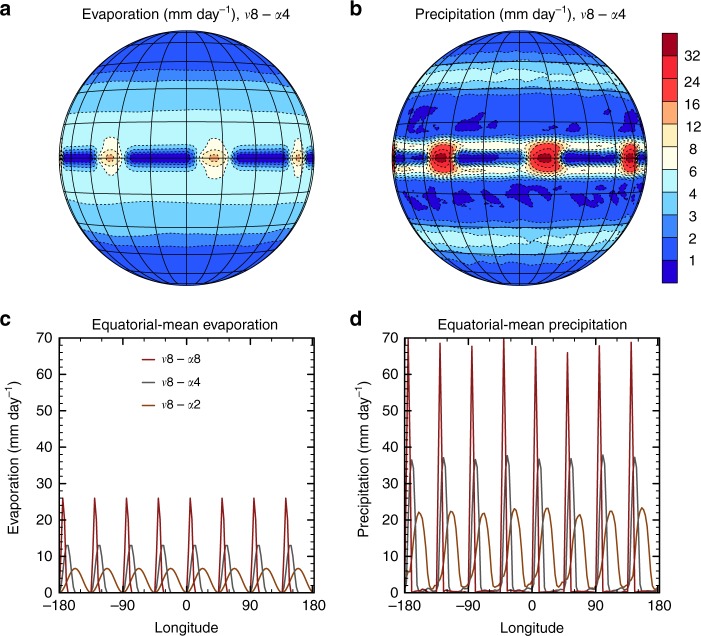
Evaporation and precipitation patterns. **a** The temporal mean evaporation in the simulation with prescribed evaporation according to a wavenumber ($\nu$) 8 pattern and an amplitude ($\alpha$) of four times the zonal-mean evaporation (at the equator). **b** The same but for the precipitation instead of the evaporation. **c** The temporal-mean evaporation as a function of longitude at the equator for the three employed amplitudes corresponding to 2, 4 and 8 times the zonal-mean value (all for wavenumber 8). Note that since the zonal-mean evaporation is the same in all simulations, a larger amplitude implies a spatial contraction of the regions of increased evaporation. **d** The same as **c** but for the temporal mean precipitation. The temporal means are taken in a statistically steady state for all displayed simulation results

To assess whether a convective clustering effect is sufficient to explain the observed relationship between the width of the ITCZ and the zonal convective clustering, we perform numerical experiments with the state-of-the-art atmospheric general circulation model IPSL-CM5A-MR (see Methods). Previous results with an idealized dry atmosphere model suggest that zonal heating patterns can cause regional changes in ITCZ^43^, which motivates us to investigate if we can even produce a zonal mean response by imposing zonal heating patterns in a model with state-of-the-art physics. We choose an idealized aquaplanet (fully water covered planet) setup with a fixed zonally uniform distribution of sea-surface temperatures to drive different states of zonal convective clustering and study how the ITCZ responds. To force the convection into the desired patterns, we prescribe zonally varying evaporation patterns such that the zonal mean is preserved (see Methods, Fig. 3 and Supplementary Fig. 1). As in observations, stronger zonal convective clustering along the equator leads to a wider ITCZ in these simulations (Fig. 2, Table 1). Since we only impose zonal changes, this shows that changes in zonal convective clustering are sufficient to drive changes in the ITCZ width.

**Fig. 4:**
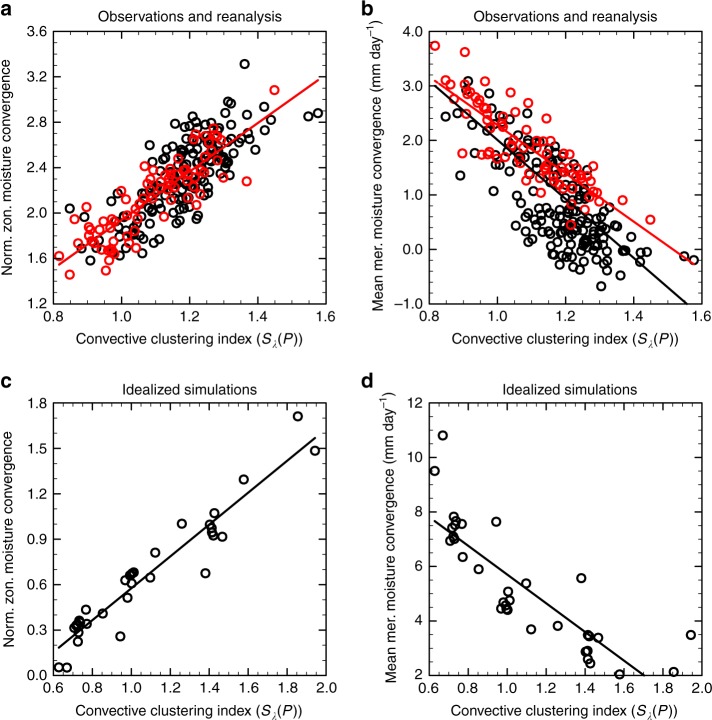
Zonal and meridional moisture convergence. **a** The standard deviations of the equatorial zonal moisture convergence from the ERA-Interim reanalysis dataset normalized by the mean precipitation at the equator as a function of convective clustering (*S*${}_{\lambda }(P)$) (from GPCP). **b** The monthly mean meridional moisture convergence from the ERA-Interim reanalysis dataset at the equator as a function of convective clustering. Red circles denote the months with a tropical precipitation distribution that is symmetric about the equator and black circles those with an asymmetric distribution. The red line is obtained by linear regression to the red circles and the black line is obtained by linear regression to all (red and black) circles. **c** The same as **a** but for a suite of model simulations associated with different zonal convective clustering with each marker representing the statistically steady-state mean value of a simulation. **d** The same as **c** but for the mean meridional moisture convergence

**Fig. 5:**
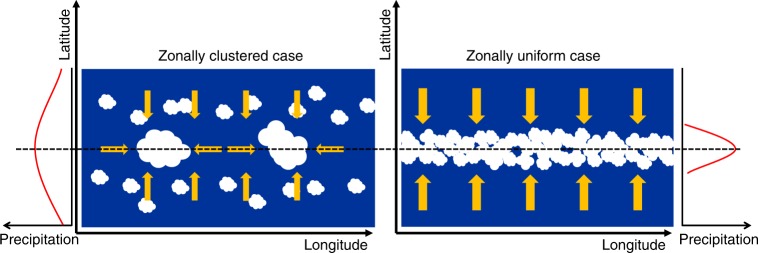
Sketch of clustered vs uniform states The sketch shows a perspective from the top of the atmosphere on the longitude-latitude plane. We indicate the regions of strong convection with white clouds over a blue (sea) surface. Yellow arrows indicate the dominating direction of moisture convergence in the boundary layer with their width indicating the strength of the flow. The dashed line corresponds to the center of the intertropical convergence zone (here to coincide with the equator). In the clustered state, the equatorial convection zones draw in moisture from all directions, and the increase of moisture convergence in zonal direction leads to a decrease in meridional moisture convergence. Therefore, more moisture is available for convection poleward of the equatorial region leading to more precipitation there, while the overall precipitation at the equator is smaller than in the zonally uniform case

The imposed zonal patterns of evaporation may also affect the atmospheric moisture budget. Since the evaporation is held approximately constant near the equator in our simulations, any change in steady-state precipitation has to be compensated by a change in moisture convergence. Since the equatorial-mean precipitation decreases with stronger convective clustering, the equatorial-mean meridional moisture convergence thus decreases as well (Fig. 4). A decrease in equatorial-mean meridional moisture convergence is also found in the daily ERA-Interim reanalysis data (Fig. 4). While the meridional convergence decreases, the contribution of the anomalous zonal moisture convergence to the equatorial precipitation increases in both the reanalysis data and simulations. This suggests that in more clustered states, a larger fraction of the moisture convergence into the convective regions occurs along the zonal direction. The increased zonal moisture convergence may thus contribute to a decrease in meridional moisture convergence as sketched in Fig. 5.

### An energetic perspective of convective clustering

**Fig. 6:**
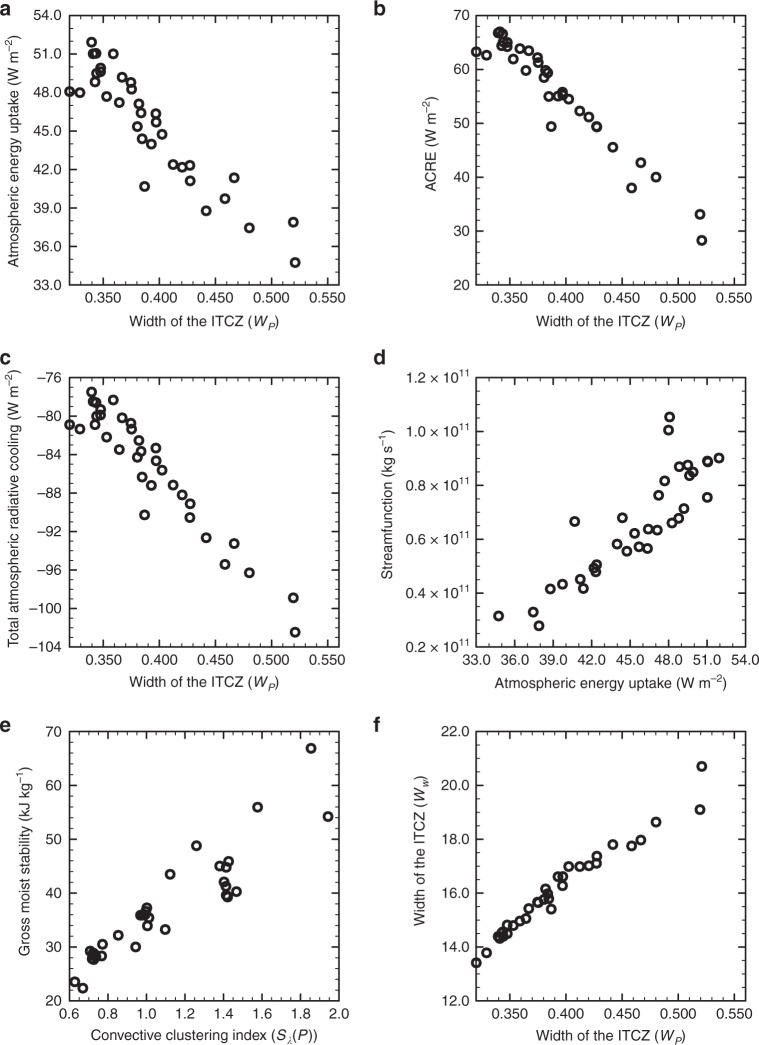
The impact of the atmospheric energy budget on the width of the intertropical convergence zone in the simulations. **a** The net atmospheric energy uptake averaged from 6 N to 6 S, **b** the atmospheric cloud-radiative effect (ACRE) and **c** the total vertically integrated radiative cooling as a function of the precipitation-inferred width of the intertropical convergence zone (ITCZ, ${W}_{P}$). **d** The Eulerian-mean mass stream function at 500 hPa and 6 degrees latitude (averaged over both hemisphere, ${\it{{\varPsi}} }_{\rm{max}}(\phi ={6}^{\rm{o}})$) as a function of the net atmospheric energy uptake averaged from 6 N to 6 S. **e** The gross moist stability at 6 degrees latitude ($\Delta h(\phi ={6}^{\rm{o}})$) as a function of the zonal convective clustering (*S*${}_{\lambda }(P)$). **f** The dynamically inferred width of the ITCZ (${W}_{\omega }$) as a function of ${W}_{P}$. In all the panels each marker corresponds to the mean value obtained from one model simulation in a statistically steady state

Several studies have shown that the behavior of the ITCZ could be explained to a large extent by energetic arguments^10–13,15–18^. Consistently, the widening of the ITCZ with the convective clustering in our simulations can be interpreted by looking at the atmospheric energy budget. The reduction of areas covered by deep convective clouds with stronger convective clustering leads to a reduction of the equatorial atmospheric cloud-radiative effect. Since the changes in atmospheric clear-sky radiative cooling and turbulent energy fluxes at the surface are small in comparison, the decrease in the equatorial atmospheric cloud-radiative effect results in a decrease in net atmospheric energy uptake (sum of the vertically integrated radiative heating and the surface turbulent fluxes) and in a weakening of the mean meridional circulation^10,11,15,18,44^ (Fig. 6). We can show this more quantitatively by following previous work^10,11,18^: Taking advantage of the statistically steady-state conditions and the hemispheric symmetry in our simulations, we can write the strength of the meridional circulation ${\it{{\varPsi}} }_{\text{max}}(\phi )$ at latitude $\phi$ as a function of the mean net atmospheric energy uptake (${\overline{{H}_{\text{a}}}}^{[0,\phi ],t}$) between the equator and latitude $\phi$, the area $A(\phi )$ from the equator to latitude $\phi$ and the gross moist stability $\Delta h$ (derived in the Methods section)

1 $${\it{{\varPsi}} }_{\text{max}}(\phi )=\frac{A(\phi ){\overline{{H}_{\text{a}}}}^{[0,\phi ],t}}{\Delta h(\phi )}\,.$$

Because the gross moist stability is positive and because we keep the considered $A(\phi )$ fixed between experiments, this equation implies that a reduction of the net atmospheric energy uptake in the equatorial region leads to a weakening of the meridional circulation for fixed latitude $\phi$. Moreover, the gross moist stability increases with clustering in our simulations (Fig. 6), implying that the gross moist stability also substantially contributes to the weakening of the Hadley circulation. A weakening of the mean-meridional circulation leads to a widening of the ascent region, due to a reduction of low-level up-gradient meridional advection of moist static energy (MSE) and to the associated widening of the low-level MSE distribution^11^. The weakening of the mean meridional circulation is thus consistent with a wider ascent region and precipitation distribution (Fig. 6). A similar framework even predicts a narrowing of the width of the dynamical ITCZ relative to the total extent of the Hadley circulation, if the contribution of the net atmospheric energy uptake from the ascending region (corresponding to the dynamical ITCZ) decreases relative to the atmospheric energy uptake of the entire Hadley circulation^10^.

Does the zonal convective clustering have the same influence on the cloud-radiative effect in observations? With the observational energy balanced and filled (EBAF) dataset of the Clouds and the Earth’s Radiant Energy System (CERES)^45–47^, we find indeed that a stronger zonal convective clustering is associated with a weaker atmospheric cloud-radiative effect and an enhanced equatorial atmospheric radiative cooling (Table 1, Supplementary Fig. 2). However, the uncertainties in the radiative fluxes from CERES-EBAF are currently too large to assess whether these relations are indeed robust (see Methods). So while these observations in principle tend to support the energetic framework outlined above, a rigorous observational verification is not yet possible. It is left to future investigations to determine whether the change in net atmospheric energy uptake with convective clustering indeed scales with the atmospheric cloud-radiative effect.

While the energetic mechanism explains the results well in the simulations, other mechanisms may also contribute to the observed link between zonal convective clustering and the width of the ITCZ. In particular, it would be interesting to see what additional insights one may gain in this regard from dynamical frameworks, such as from Matsuno-Gill type models^31,43,48–51^. Such models could be especially useful to study the dynamic response associated with changing the zonal wavenumber on the one hand and with changing the amplitude of the imposed evaporation forcing on the other hand. In our simulations, the amplitude has the largest effect on the zonal convective clustering (Supplementary Table 2), with an increase in amplitude leading to a widening of the ITCZ and a reduction of equatorial precipitation, but we will leave the determination of the involved mechanisms to future studies.

As mentioned earlier the widening of the ITCZ with stronger zonal convective clustering also tends to be associated with a more pronounced double-peak structure of the ITCZ. The transition from a single to a double ITCZ with a weaker circulation can be explained by the decreasing magnitude of the mean meridional circulation relative to the turbulent surface energy fluxes: The turbulent energy fluxes have two maxima off the equator, where the trades are stronger, and thus favor two peaks in the low-level MSE structure^52^, whereas a strong mean-meridional circulation favors one peak due to strong upgradient meridional advection of MSE^11,53^. Therefore, a weakening of the mean-meridional circulation does not only lead to a widening of the ITCZ but also favors a double ITCZ structure due to the increased importance of the turbulent surface energy fluxes in setting the zonal-mean meridional low-level MSE distribution. Whether the link between the width of the ITCZ and the double-peak structure can also be explained by alternative arguments, will have to be explored in the future.

## Discussion

Changes in the partitioning between zonal and meridional convergence have been observed during the El-Niño Southern Oscillation (ENSO)^25,54^ and were found in aquaplanet simulations with zonal heterogeneities in tropical surface temperatures^55^. The zonally oriented Walker circulation and the meridionally oriented Hadley circulation are anti-correlated, with a weaker (stronger) Walker (Hadley) circulation during El Niño events^25,54^. The meridional circulation also weakens and the zonal circulation strengthens when zonal asymmetries are introduced in simulations^55^. Our observational results are consistent with these findings, although ENSO (evaluated with the Nino 3.4 index) explains only 11% of the variance in zonal convective clustering, 32% of the variance in the equatorial mean precipitation and 21% of the variance in the width of the ITCZ. ENSO is thus not the main contributor to the observed variations.

**Fig. 7:**
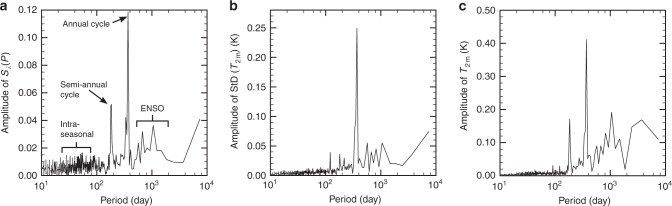
Temporal spectra. **a** The amplitude of the temporal Fourier transform for the zonal convective clustering ${S}_{\lambda }(P)$, **b** for the zonal standard deviation of the 2m-temperature between 6 S and 6 N (StD(${T}_{\text{2m}}$)) and **c** for the average 2m-temperature (${T}_{\text{2m}}$) between 6 S and 6 N as a function of the corresponding period. The largest peak corresponds to a period of one year and the second largest to half a year. The maximum at around a 1000 days corresponds approximately to the period of the El Niño Southern Oscillation (ENSO). The maximum at the longest period indicates a slight trend in ${S}_{\lambda }(P)$

This raises the question as to whether additional modes of natural variability could explain the variance in convective clustering. A spectral analysis of the zonal convective clustering suggests a strong signal from the annual and semi-annual cycles (Fig. 7), a weak ENSO signal and almost no signal from the MJO (less than 5% of the variance in clustering explained). To investigate how much of the link between zonal convective clustering and the ITCZ width comes from the annual cycle, we remove the annual cycle from the zonal convective clustering, from the equatorial precipitation and from the width of the ITCZ and find the variance explained between these variables to decrease by about half. However, a substantial correlation between the zonal convective clustering and the width of the ITCZ is also present in selected months (Supplementary Table 3), showing that the annual cycle is not the sole driver of the relationship between the convective clustering and the width of the ITCZ.

The annual and semi-annual cycles do not only influence the zonal convective clustering but also the 2m temperatures in the equatorial region (Fig. 7), and we find a high anti-correlation between the tropical 2m temperatures and zonal convective clustering (Table 1). Changes in meridional near-surface temperature gradients could also lead to changes in the ITCZ width. So the high anti-correlation between zonal convective clustering and the deep tropical 2m temperature raises the question whether the changes in meridional near-surface temperature gradients could be driving both the changes in zonal convective clustering and the changes in ITCZ width. We find that this is not the case, since the zonal convective clustering is not associated with systematic changes in meridional near-surface temperature gradients (Table 1). Instead, stronger zonal convective clustering is favored by larger zonal temperature gradients (Table 1).

In summary, this study shows that there is a strong relationship between the zonal convective clustering and the meridional distribution of rainfall in the topics. At times of stronger clustering, the ITCZ widens, which reduces the mean precipitation close to the equator and promotes rainfall at higher tropical latitudes. Furthermore, the mean-meridional convergence decreases while the zonal circulations increase both in observations and in simulations. Our simulations show that the change in ITCZ width can be caused by changes in zonal clustering of convection, and not the opposite. They also suggest that stronger zonal convective clustering decreases the cloud-radiative heating of the equatorial atmosphere thus leading to a smaller meridional gradient of net atmospheric energy uptake which weakens the meridional circulation and leads to a widening of the ITCZ. The largest changes in the zonal clustering found in observations are partly driven by zonal variations in surface temperature, in particular those related to the seasonal cycle, but our idealized aquaplanet simulations suggest that for a given surface temperature distribution the zonal clustering itself amplifies these effects (Table 1). The substantial impact of the zonal clustering of convection on the tropical rainfall distribution suggests that the ability of models to represent the zonal convective clustering along the equator is of primary importance in the quest for more realistic predictions of the tropical rain belt. In particular, our study suggests that potential model biases in the zonal convective clustering could contribute to known biases in the width^15^ and the double peak structure^56,57^ of the ITCZ.

## Methods

### Indicators and observational and reanalysis data

We use the Global Precipitation Climatology Project (GPCP) daily data with a resolution of 0.75° × 0.75° for the period from October 1997 to December 2016^37,38^ interpolated on a 1° × 1° lat-lon grid. We use this data to calculate the zonal-mean precipitation, the relative width of the precipitation-inferred ITCZ (${W}_{P}$) and the different metrics for the zonal clustering of convection. The relative width of the precipitation is defined as

2 $${W}_{P}=\frac{{\overline{P}}^{15\mathrm{S}-15\mathrm{N}}}{{\overline{P}}^{6\mathrm{S}-6\mathrm{N}}}$$

where $P$ is the precipitation and overlines denote means over the zonal bands between the specified latitudes. ${W}_{P}$ is positive and increases with decreasing contribution of the band from 6 S to 6 N to the deep tropical mean precipitation and hence with a wider ITCZ.

We use a second metric to identify the width of the ITCZ namely the meridional extent of the region for which the zonal-mean vertical pressure velocity ($\omega$) at 500 hPa is smaller than zero (${W}_{\omega }$) around the maximum zonal-mean precipitation, indicating mean ascent^10,12,15,18^. Since this also corresponds to the distance between the two maxima of the mean-meridional Eulerian mass-stream function at 500 hPa, this metric can be considered as a dynamical width of the ITCZ. The pressure velocity is obtained from the European Center for Medium-Range Weather Forecasts Interim Reanalysis (ERA-interim)^41^ dataset from October 1996 to December 2016, by interpolating onto a lat-lon grid with ${1}^{\rm{o}}{\rm{\times}}{1}^{\rm{o}}$ resolution and taking daily means of the 6 hourly data. Note that since there is only one tropical ascent region in the monthly and zonal mean ERA-interim data (even if there are two peaks in precipitation), the value calculated for ${W}_{\omega }$ is not dependent on the definition used to infer the ITCZ position.

In order to assess whether the precipitation is symmetric about the equator we use the tropical precipitation asymmetry index^8^:

3 $${A}_{P}=\frac{{\overline{P}}^{0\mathrm{S}-15\mathrm{N}}-{\overline{P}}^{15\mathrm{S}-0\mathrm{S}}}{{\overline{P}}^{15\mathrm{S}-15\mathrm{N}}},$$

and define the precipitation distribution to be hemispherically symmetric if $|{A}_{P}| \, < \, 0.4$. With this definition around a third of all considered months are symmetric and all symmetric months lie between November and May. The months that are most often symmetric are December and April. Note that the differences between months with symmetric and asymmetric precipitation vanish for threshold value of $|{A}_{P}|$ between $0.45$ and $0.50$ with values below $0.45$ leading to the distinct differences presented in this manuscript.

To characterize the zonal convective clustering we use the monthly averaged daily zonal standard deviation of the meridionally averaged precipitation from 6 S to 6 N, normalized by the mean precipitation over that region:

4 $${S}_{\lambda }(P)=\frac{{\overline{\sqrt{\frac{1}{n-1}{\sum }_{{\lambda }_{\text{i}}}{\left({\overline{P(t,{\lambda }_{\text{i}})}}^{\phi =6\mathrm{S}-6\mathrm{N}}-{\overline{P(t)}}^{\lambda ,\phi =6\mathrm{S}-6\mathrm{N}}\right)}^{2}}}}^{t}}{{\overline{P}}^{6\mathrm{S}-6\mathrm{N}}},$$

where $\lambda$ denotes longitude, $\phi$ denotes latitude, $n$ the number of grid-points in zonal direction and $t$ time. Overlines denote averages over the quantity denoted to the right.

We define ${S}_{\lambda }(P)$ in this way for several reasons. First, to avoid catching meridional shifts of the ITCZ with ${S}_{\lambda }(P)$, we choose to take a meridional average from 6 S to 6 N before calculating the zonal standard deviation of the precipitation. So ${S}_{\lambda }(P)$ describes a purely zonal signal. The zonal standard deviation of precipitation can be expected to increase with the mean precipitation. To avoid such a dependence on the mean precipitation, we choose to divide the zonal standard deviation of precipitation by the mean precipitation. However, this division by the mean precipitation could by itself also lead to a dependency of ${S}_{\lambda }(P)$ on the mean precipitation. To verify whether this is the case, we compare it with two other metrics that also quantify the spatial concentration of rainfall, but that are independent of the mean precipitation: The subsidence fraction (${F}_{w500> 0}$)^39^ in the region from 6S to 6N and the minimum area fraction that encompasses 80% of the precipitation (${F}_{\text{0.8}\overline{P}}$) in the same region.

${F}_{\text{0.8}\overline{P}}$ is obtained by sorting the daily meridionally averaged precipitation values (obtained from GPCP) from 6 S to 6 N by magnitude for each month. The meridional averaging leads again to a purely zonal signal. We then count the number of points in time and longitude space necessary to reach 80 % of the total precipitation over the month in the region, when going from strong to weak precipitation. The resulting value is finally divided by the total amount of points in time and longitude space. ${F}_{\text{0.8}\overline{P}}$ has the advantage over ${S}_{\lambda }(P)$ not to include the mean precipitation in its calculation. But since ${S}_{\lambda }(P)$ is more readily comparable to zonal anomalies in surface temperature and other quantities than ${F}_{\text{0.8}\overline{P}}$ and since ${S}_{\lambda }(P)$ is highly correlated to ${F}_{\text{0.8}\overline{P}}$ anyway (Supplementary Table 4), we choose to use ${S}_{\lambda }(P)$. Note that ${F}_{w500> 0}$ is also highly correlated with ${S}_{\lambda }(P)$ and ${F}_{\text{0.8}\overline{P}}$ (Supplementary Table 4) and thus all three metrics represent the degree of zonal convective clustering.

We select the region of 6 S to 6 N to calculate the zonal clustering as a compromise between including the center of the zonal-mean meridional precipitation distribution in as many months as possible and keeping the meridional extent of the region small enough that the meridional averaging does not smooth too much of the zonal signal by including regions of subsidence in the margins. For example, using the region from 11 S to 11 N to calculate ${S}_{\lambda }(P)$ leads qualitatively to the same picture in the symmetric months, but the correlations between the ${S}_{\lambda }(P)$ and the variables in Table 1 are smaller, because the spread in ${S}_{\lambda }(P)$ calculated from 11 S 11 N is about half of the one obtained when ${S}_{\lambda }(P)$ is calculated from 6 S to 6 N.

The vertically integrated atmospheric radiative cooling is derived from the EBAF dataset of the CERES^45–47^ from March 2000 to December 2016. It is calculated as the difference between the monthly averaged net longwave plus shortwave fluxes at the top-of-the-atmosphere and the surface. The clear-sky atmospheric heating and the atmospheric cloud radiative heating are computed consistently by using either the clear-sky or the cloud radiative effect components of the top-of-the-atmosphere and surface components. The monthly CERES-EBAF data set at ${1}^{\rm{o}}{\rm{\times}}{1}^{\rm{o}}$ resolution is estimated to have a regional uncertainty of $2.5\ \mathrm{W}{\mathrm{m}}^{-2}$ for the top-of-the-atmosphere radiative fluxes over the period from July 2002 to June 2015^46^. The uncertainty of the CERES-EBAF global annual-mean surface radiative fluxes was estimated to be $8\ \mathrm{W}{\mathrm{m}}^{-2}$^47^. These uncertainties are too large to robustly identify a signal (for the atmospheric cloud-radiative effect and the net atmospheric energy uptake) with an estimated spread of about $15\ \mathrm{W}{\mathrm{m}}^{-2}$ (Supplementary Fig. 2).

### Climate model

We perform simulations with the version 5A of the atmospheric component of the Institute Pierre Simon Laplace (IPSL) coupled model. We use the medium resolution version of the model (MR) that took part in the climate model intercomparison project phase 5 (CMIP5) under the acronym IPSL-CM5A-MR^58–60^. The atmospheric general-circulation model solves the primitive equations on a latitude-longitude Arakawa C-grid with a horizontal resolution of $1.2{5}^{\text{o}}\ {\rm{\times}}\ 2.5{3}^{\text{o}}$ in latitude × longitude. The model uses hybrid sigma pressure coordinates in the vertical and resolves the atmosphere with 39 levels up to about 70 km height. The model physics include sub-grid parameterizations for the radiative transfer^61^, turbulence and boundary layer dynamics^62,63^, cumulus convection^64,65^ and clouds^60,66^. Detailed description of the model and its performance is given in refs. ^58,60^ and ^59^.

### Boundary conditions

We perform simulations in an aquaplanet configuration, i.e. without land nor sea-ice. We assume perpetual equinox conditions, assuming a daily cycle but no seasonal cycle, and a total solar irradiance that is equivalent to $1366\ \mathrm{W}{\mathrm{m}}^{-2}$. This allows us to ascertain that the results are not caused by the seasonal cycle. The radius, dry mass of the atmosphere, the acceleration from gravity and the inertial forces are prescribed to be equal to present-day Earth. We prescribe the sea-surface temperatures that are zonally uniform but exhibit meridional gradients to mimic Earth’s mean meridional temperature gradients following the control distribution used by ref. ^67^. Non-condensable greenhouse gases are fixed to the following concentrations: 1650 ppbv for ${{\rm{CH}}}_{4}$, 306 ppbv for ${{\rm{N}}}_{2}$O, and 348 ppmv for ${{\rm{CO}}}_{2}$. In all these aspects (except for the use of the “control” sea-surface temperature distribution instead of QOBS) we follow the simple aquaplanet experiment (APE) protocol^67,68^.

### Evaporation patterns

It has been shown that convection tends to localize over maxima of boundary-layer MSE^11,52,69,70^. Therefore, we control the spatial organization of convection by imposing evaporation patterns. Regions of increased evaporation and hence enhanced latent heat flux receive an increased influx of MSE. We thereby push the maxima of MSE in the boundary layer into the regions of increased evaporation and thus convection preferably localizes there (Fig. 3 and Supplementary Fig. 1). In principle, we could force different convective patterns with different patterns of surface temperatures, but using the evaporation has the advantage of controlling the moisture flux into the atmosphere directly. Furthermore, the observed zonal convective clustering and the observed zonal evaporation clustering (calculated in the same way as the zonal convective clustering but using evaporation from ERA-interim instead of precipitation) are highly correlated with a correlation coefficient of 0.68 (0.84 in the case of a symmetric precipitation distribution about the equator).

In all but the control simulation, the zonal distribution of evaporation is prescribed within a zonal band from 10 S to 10 N. At higher latitudes the evaporation evolves freely. We choose to vary two parameters of the evaporation patterns: The total area of the regions with increased evaporation, and the distribution of these regions. We focus on the total convective area, as this is what we focused on in the observations. However, a given total convective area can be associated with different numbers of convective areas. To investigate the relative influence of the total area versus the number of convective areas we perturb the evaporation fields in two ways: such that the total area covered by convection varies and such that the total number of convective regions varies. The forced evaporation fields all have the same zonal means as in the control simulation but vary periodically in zonal direction. An example of evaporation fields is shown in Fig. 3. We define the imposed evaporation using the following function

5 $$E(\phi ,\lambda )=\left\{\begin{array}{cc}\frac{{E}_{\text{max}}(\phi )+{E}_{\text{min}}(\phi )}{2}+\frac{{E}_{\text{max}}(\phi )-{E}_{\text{min}}(\phi )}{2}\cdot \text{sin}\left(\frac{\alpha \nu }{2}\lambda -90\right),&\text{if}\ \text{mod}\left(\lambda ,\frac{360}{\nu }\right)\le \frac{2\cdot 360}{\alpha \nu } \\ {E}_{\text{min}}(\phi ),&\text{otherwise}\end{array}\right.$$

where $\lambda$ denotes the longitude, $\phi$ the latitude, $E$ the evaporation, $\nu$ the imposed zonal wavenumber and $\text{mod}$(*a*,*b*) denotes the real-valued reminder of the division of $a$ by $b$. The other quantities are defined as

6 $$\begin{array}{ccc}&&\alpha :=\frac{{E}_{\text{max}}(\phi =0)}{{\overline{{E}_{\text{c}}}}^{\lambda }(\phi =0)}\\ &&{E}_{\text{min}}(\phi )={\overline{{E}_{\text{c}}}}^{\lambda }(\phi )\cdot |\frac{\phi }{{\phi }_{\text{l}}}|\end{array}$$

$${E}_{\text{max}}(\phi )=\alpha \cdot {\overline{{E}_{\text{c}}}}^{\lambda }(\phi )-(\alpha -1)\cdot {E}_{\text{min}}(\phi )$$

where ${\overline{{E}_{\text{c}}}}^{\lambda }(\phi )$ denotes the zonal-mean evaporation from the control simulation and ${\phi }_{\text{l}}$ the latitude at which the amplitude of the zonal oscillation is zero. For any given ${\overline{{E}_{\text{c}}}}^{\lambda }(\phi )$, $\alpha \ge 2$, $\nu \in {\mathbb{N}}$ and ${\phi }_{\text{l}}$ the evaporation field is well defined, smooth and stationary. Furthermore, the zonal-means of the thus defined fields correspond to the zonal-mean evaporation from the control simulation at all latitudes (${\overline{E}}^{\lambda }(\phi )={\overline{{E}_{\text{c}}}}^{\lambda }(\phi )$). For $\alpha =2$ the evaporation is proportional to the sine of longitude. For $\alpha \, > \, 2$ the sinusoid oscillations are interrupted by regions of constant evaporation (in zonal direction). The periods of the oscillations shorten with increasing $\alpha$ in order to keep the zonal mean constant. The amplitude of the oscillation decreases with latitude until it is zero at latitude ${\phi }_{\text{l}}$. We use ${\phi }_{\text{l}}=10$ in all experiments. Note that the standard deviation of the thus defined evaporation at the equator is independent of the zonal wavenumber. We thus expect that the zonal convective clustering (evaluated based on $S_{\lambda }(P)$) depends mostly on $\alpha$.

We relax the evaporation against the aforementioned field in a zonal band from 10 S to 10 N using Newtonian relaxation with

7 $${E}_{\text{m}}(t+\Delta t,\lambda ,\phi )=\widehat{E}(t+\Delta t,\lambda ,\phi )+(E(\lambda ,\phi )-\widehat{E}(t+\Delta t,\lambda ,\phi ))\cdot \frac{\Delta t}{\tau (\phi )},$$

where $t$ is time, $\Delta t$ is the model time-step, $\widehat{E}$ is the evaporation calculated by the surface scheme, ${E}_{\text{m}}$ is the actual evaporation seen by the model physics and $\tau (\phi )$ is the latitude-dependent relaxation constant. We model $\tau (\phi )$ according to

8 $$\tau (\phi )={\tau }_{\text{n}}\cdot \left|\frac{\phi }{{\phi }_{\text{n}}}\right|,$$

where ${\tau }_{\text{n}}$ is the relaxation time step at latitude ${\phi }_{\text{n}}$. This means that the relaxation is strongest at the equator and decreases with latitude. If $\tau (\phi )\le \Delta t$ the evaporation is prescribed to the field $E(\lambda ,\phi )$. This is effectively always the case at the equator. In all the experiments (except the control) we use ${\tau }_{\text{n}}=3600\ \mathrm{s}$ and ${\phi }_{\text{n}}=10$.

### Experiments

We perform experiments for a range of $\alpha$ and $\nu$. These are all run for a total of 1080 (3 times 360) days. The first 360 days are discarded for spin-up noise, which leaves 720 days in a statistically steady state to analyze. In the control experiment (henceforth CTRL) the evaporation is unforced. The state of CTRL after 360 days is also used as initial condition to start the other experiments. ${\overline{{E}_{\text{c}}}}^{\lambda }(\phi )$ corresponds to the zonal-mean evaporation averaged from day 361 to 1080 in CTRL. In one experiment we force a zonally uniform evaporation at the value from ${\overline{{E}_{\text{c}}}}^{\lambda }(\phi )$, using the relaxation as specified in the previous section. In the other experiments, we apply different values of $\nu$ and $\alpha$ to Eqs. (5) and (6) to create a large range of evaporation fields as explained in the previous subsection. We use the values 1 through 10 for $\nu$ and 2, 4 and 8 for $\alpha$ which leads to $30=10\cdot 3$ experiments as well as two experiments with $\nu =20$ and $\alpha =2,4$.

### Model diagnostics

We use the same diagnostics to analyze the model simulations as for the observational and reanalysis datasets, except that we consider the daily values for the entire statistically steady-state period, instead of single months. Hence, whenever a scatter plot with model output is shown, one marker corresponds to one of the simulations.

### Intensity of meridional circulation and energy transport

In order to link the intensity of the circulation to the meridional energy transport, we start from the energy balance equation in flux form^10,11,18^:

9 $${F}_{\text{H}}(\phi ,t)-{F}_{\text{H}}(-\phi ,t)=\int _{{\phi }{{\prime} }=-\phi }^{{\phi }{{\prime} }=\phi }\int _{0}^{2\pi }\left({H}_{\text{a}}(\lambda ,\phi {\prime} ,t) + \left\langle \ {\frac{\partial e(\lambda ,\phi {\prime} ,t)}{\partial t}} \right\rangle _{\text{p}}\right){\text{cos}(\phi {\prime} )}{a}^{2}\mathrm{d}\lambda \mathrm{d}\phi {\prime} ,$$

where $\phi$ denotes the latitude, $\lambda$ the longitude, $t$ time, $a$ the radius of the Earth, ${H}_{\text{a}}$ the net atmospheric energy uptake, that is the sum of the vertically integrated radiative heating and the turbulent surface energy fluxes and ${F}_{\text{H}}(\phi ,t)$ the total meridional energy flux across latitude $\phi$. ${\langle \cdot \rangle }_{\text{p}}$ denotes the mass weighted vertical integral over the entire atmosphere and $e$ the total energy, that is the sum of the kinetic energy and the frozen MSE $h$ defined as

10 $$h={c}_{\text{p}}T+gz+Lq-{L}_{\text{f}}{q}_{\text{i}},$$

where ${c}_{\text{p}}$ is the specific heat capacity of air at constant pressure, $T$ the temperature, $g$ Earth’s gravity acceleration, $z$ the height above sea level, $L$ the latent energy of vaporization, ${L}_{\text{f}}$ the latent heat of fusion, $q$ the specific humidity and ${q}_{\text{i}}$ the specific mass of ice.

In a statistically steady state, the temporal derivative vanishes when averaged over sufficiently long time periods, and Eq. (9) simplifies to

11 $${\overline{{F}_{\text{H}}(\phi )}}^{t}-{\overline{{F}_{\text{H}}(-\phi )}}^{t}=\int _{\phi {\prime} =-\phi }^{\phi {\prime} =\phi }\int _{0}^{2\pi }{\overline{{H}_{\text{a}}(\lambda ,\phi {\prime} )}}^{t}{\text{cos}(\phi {\prime} )}{a}^{2}{\mathrm{d}}\lambda {\mathrm{d}}\phi {\prime} ,$$

where ${\overline{\cdot }}^{t}$ denotes the temporal mean. In the aquaplanet simulations we can assume the two hemispheres to be symmetric. Therefore, the two terms on the left hand side are equal in magnitude and opposite in sign and the integral on the right hand side is twice the integral with bounds from latitudes 0 to $\phi$. Hence we can rewrite Eq. (12) as

12 $${\overline{{F}_{\text{H}}(\phi )}}^{t}=\int _{0}^{\phi {\prime} =\phi }\int _{0}^{2\pi }{\overline{{H}_{\text{a}}(\lambda ,\phi {\prime} )}}^{t}{\text{cos}(\phi {\prime} )}{a}^{2}{\mathrm{d}}\lambda {\mathrm{d}}\phi {\prime} \,.$$

Defining the total vertical energy flux into the atmosphere on the right-hand side as $H(\phi )$

13 $${\overline{H(\phi )}}^{t}:= \int_{0}^{\phi {\prime} =\phi } \int _{0}^{2\pi }{\overline{{H}_{\text{a}}(\lambda ,\phi {\prime} )}}^{t}{\text{cos}(\phi {\prime} )}{a}^{2}{\mathrm{d}}\lambda {\mathrm{d}}\phi {\prime} ,$$

we obtain

14 $${\overline{{F}_{\text{H}}(\phi )}}^{t}={\overline{H(\phi )}}^{t}\,.$$

${\overline{{F}_{\text{H}}(\phi )}}^{t}$ can be rewritten using the meridional wind $v$ and the frozen MSE $h$ as

15 $${\overline{{F}_{\text{H}}(\phi )}}^{t}=2\pi a{\text{cos}(\phi )} \langle {\overline{h(\phi )v(\phi )}}^{\lambda ,t} {\rangle }_{\text{p}},$$

where ${\overline{\cdot }}^{\lambda ,t}$ denotes the zonal and temporal means.

We now introduce the gross moist stability in a similar way as in the original work on the subject^44^:

16 $${\overline{\Delta h(\phi )}}^{t}=\frac{\langle {\overline{h(\phi )v(\phi )}}^{\lambda ,t} {\rangle }_{\text{p}}}{\langle {\overline{v(\phi )}}^{\lambda ,t} {\rangle }_{{\text{p}}_{\text{m}}}},$$

where $\langle \cdot {\rangle }_{{{p}}_{\text{m}}}$ denotes the mass weighted vertical integral from the top of the atmosphere to a mid level ${p}_{\rm{m}}$ where the meridional wind changes direction. Applying this definition of the gross moist stability to Eq. (15) yields

17 $${\overline{{F}_{\text{H}}(\phi )}}^{t}={\overline{\Delta h(\phi )}}^{t}\langle 2\pi a{\text{cos}(\phi )}{\overline{v(\phi )}}^{\lambda ,t} {\rangle }_{{\text{p}}_{\text{m}}}\,.$$

We note that

18 $${\overline{{\it{{\varPsi}} } (\phi ,{p}_{\text{m}})}}^{t}=\langle 2\pi a{\text{cos}(\phi )}{\overline{v(\phi )}}^{\lambda ,t} {\rangle }_{{{p}}_{\text{m}}},$$

where ${\it{\Psi}} (\phi ,{p}_{\text{m}})$ is the Eulerian-mean meridional mass stream function and obtain

19 $${\overline{{F}_{\text{H}}(\phi )}}^{t}={\overline{\Delta h(\phi )}}^{t}{\overline{{\it{{\varPsi}} } (\phi ,{p}_{\text{m}})}}^{t}\,.$$

So far the only assumptions we have made are a hemispheric symmetry and a statistically steady state. In particular, the definition of the gross moist stability here takes the energy advection due to eddies into account. We will now also assume that the mid level ${p}_{\text{m}}$ is at 500 hPa, and that this is the level where the meridional wind changes direction. This is equivalent to assuming that the streamfunction also maximizes at 500 hPa and we thus approximate

20 $${\overline{{\it{{\varPsi}} }_{\text{max}}(\phi )}}^{t}\approx {\overline{{\it{{\varPsi}} } (\phi ,{p}_{\text{m}}=500\ \text{hPa})}}^{t}\,.$$

Since the mean meridional wind is typically weak in the mid troposphere, the exact choice of the mid level does not substantially affect the results^44^. Applying Eqs. (14) and (20) to Eq. (19) and solving for ${\it{\Psi }}_{\text{max}}(\phi )$ finally yields

21 $${\overline{{\it{{\varPsi}} }_{\text{max}}(\phi )}}^{t}=\frac{{\overline{H(\phi )}}^{t}}{{\overline{\Delta h(\phi )}}^{t}}\,.$$

We note that ${H}_{\text{a}}$ averaged over the surface area enclosed by the latitudes 0 and $\phi$ is

22 $${\overline{{H}_{\text{a}}}}^{[0,\phi ],t}=\frac{{\int }_{0}^{\phi {\prime} =\phi }{\int }_{0}^{2\pi }{\overline{{H}_{\text{a}}(\lambda ,\phi {\prime} )}}^{t}{\text{cos}(\phi {\prime} )}{a}^{2}\mathrm{d}\lambda \mathrm{d}\phi {\prime} }{{\int }_{0}^{\phi {\prime} =\phi }{\int }_{0}^{2\pi }{\text{cos}(\phi {\prime} )}{a}^{2}\mathrm{d}\lambda \mathrm{d}\phi {\prime} }\,.$$

If we denote the surface area enclosed by the latitudes 0 and $\phi$ with

23 $$A(\phi )=\int _{0}^{\phi {\prime} =\phi }\int _{0}^{2\pi }{\text{cos}({\phi }{{\prime} })}{a}^{2}\mathrm{d}\lambda \mathrm{d}{\phi }{{\prime} }$$

we can write

24 $${\overline{{H}_{\text{a}}}}^{[0,\phi ],t}=\frac{{\overline{H(\phi )}}^{t}}{A(\phi )}\,.$$

Finally we can rewrite Eq. (21) using Eq. (24) as

25 $${\it{{\varPsi}} }_{\text{max}}(\phi )=\frac{A(\phi ){\overline{{H}_{\text{a}}}}^{[0,\phi ],t}}{{\overline{\Delta h(\phi )}}^{t}}\,.$$

## Supplementary Information

Supplementary Information

## Data Availability

The processed GPCP data is available on the website of the Observations for Model Intercomparisons Project (obs4MIPs, https://esgf-node.llnl.gov/projects/obs4mips/). The ERA-interim data is available upon registration on the homepage of ECMWF (https://www.ecmwf.int/en/forecasts/datasets/reanalysis-datasets/era-interim). The processed data used here is available on IPSL’s ClimServ http://climserv.ipsl.polytechnique.fr/fr/les-donnees/era-interim.html upon request. The ERA-5 data is available through Copernicus at https://cds.climate.copernicus.eu. The amplitude of the MJO is available at Columbia University at https://iridl.ldeo.columbia.edu/SOURCES/.BoM/.MJO/.RMM/.amplitude/. The monthly Niño 3.4 SST index inferred from the HadISST1 data set is available at NOAA at https://www.esrl.noaa.gov/psd/gcos_wgsp/Timeseries/Nino34/. The processed CERES-EBAF data is available on IPSL’s ClimServ at http://climserv.ipsl.polytechnique.fr/cfmip-obs/. The original data can be obtained at NASA at https://ceres.larc.nasa.gov/. All formated data for the analysis performed here can be obtained from the authors upon request. Due to the size of the datasets, the simulation results can only be obtained from the authors upon request.

## Code availability

The atmospheric component of the IPSL-5A-MR climate model LMDZ (to be run in version 5A) used in this study is freely available at http://svn.lmd.jussieu.fr/LMDZ/LMDZ6/trunk. The specific version of the model code that allows the prescription of the evaporation patterns can be obtained from the authors along with all necessary boundary conditions for the specific simulations and all scripts used to process the simulation results.
