# Author Correction: Stronger zonal convective clustering associated with a wider tropical rain belt

**DOI:** 10.1038/s41467-019-13645-w

**Published:** 2019-12-04

**Authors:** Max Popp, Sandrine Bony

**Affiliations:** Laboratoire de Météorologie Dynamique (LMD/IPSL), Sorbonne Université, Centre National de la Recherche Scientifique (CNRS), École Polytechnique, École Normale Supérieure, 4 Place Jussieu, 75005 Paris, France

**Keywords:** Atmospheric science, Atmospheric dynamics, Climate change, Climate and Earth system modelling

Correction to: *Nature Communications* 10.1038/s41467-019-12167-9, published online 19 September 2019.

The original version of this Article contained errors in Fig. 1. In Fig. 1a, the label at the top of the left panel incorrectly read ‘*S*_*λ*_(*P*) = 1.45, *W*_*P*_ = 1.08 and *W*_*ω*_ = 32.3’. The correct version reads ‘*S*_*λ*_(*P*) = 0.90, *W*_*P*_ = 0.71 and *W*_*ω*_ = 25.3’. Similarly in Fig. 1b, the label at the top of the left panel incorrectly read ‘*S*_*λ*_(*P*) = 0.90, *W*_*P*_ = 0.71 and *W*_*ω*_ = 25.3’. The correct version reads ‘*S*_*λ*_(*P*) = 1.45, *W*_*P*_ = 1.08 and *W*_*ω*_ = 32.3’. This has been corrected in both the PDF and HTML versions of the Article.

