# Supplementary material for: Stronger zonal convective clustering associated with a wider tropical rain belt
Source: Nat Commun. 2019 Sep 19;10:4261. doi: 10.1038/s41467-019-12167-9 (PMC6753108; doi:10.1038/s41467-019-12167-9)
Supplement: Supplementary file 1 — Supplementary Information [file 41467_2019_12167_MOESM1_ESM.pdf]

Supplementary Information

Stronger zonal convective clustering associated with a wider tropical rain belt

Max Popp[1⋆] and Sandrine Bony[1]

[1] Laboratoire de Météorologie Dynamique (LMD/IPSL), Sorbonne Université, Centre National de la Recherche Scientifique (CNRS), École Polytechnique, École Normale Supérieure, 4 Place Jussieu, 75005 Paris, France

⋆ email: max.popp@lmd.jussieu.fr

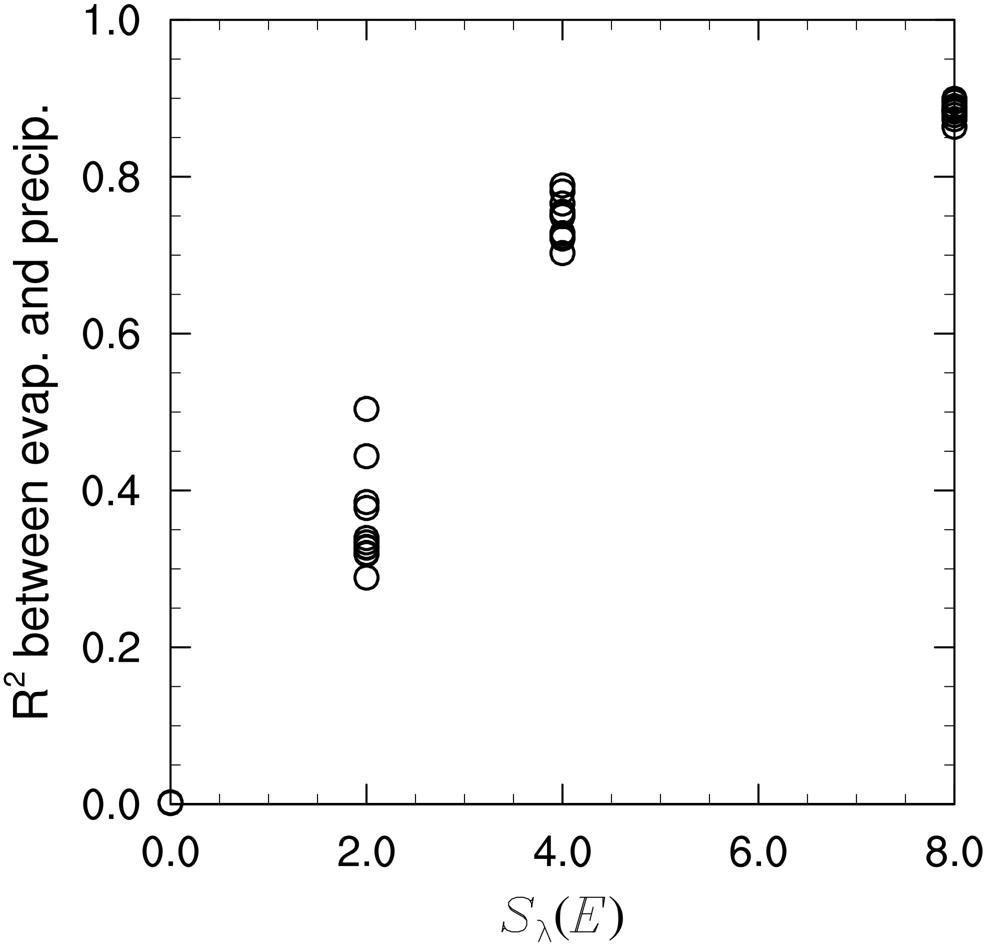

Supplementary Figure 1 | Spatial correlation between evaporation and precipitation. The figure shows the spatial and daily correlation coefficient squared between the meridional mean evaporation and the precipitation between 6 S and 6 N as a function of the amplitude of the imposed evaporation forcing (at the equator) normalized by the equatorial mean ($S_\lambda(E)$). The two simulations with amplitude 0 are the control simulation and the simulation with no zonal variations.

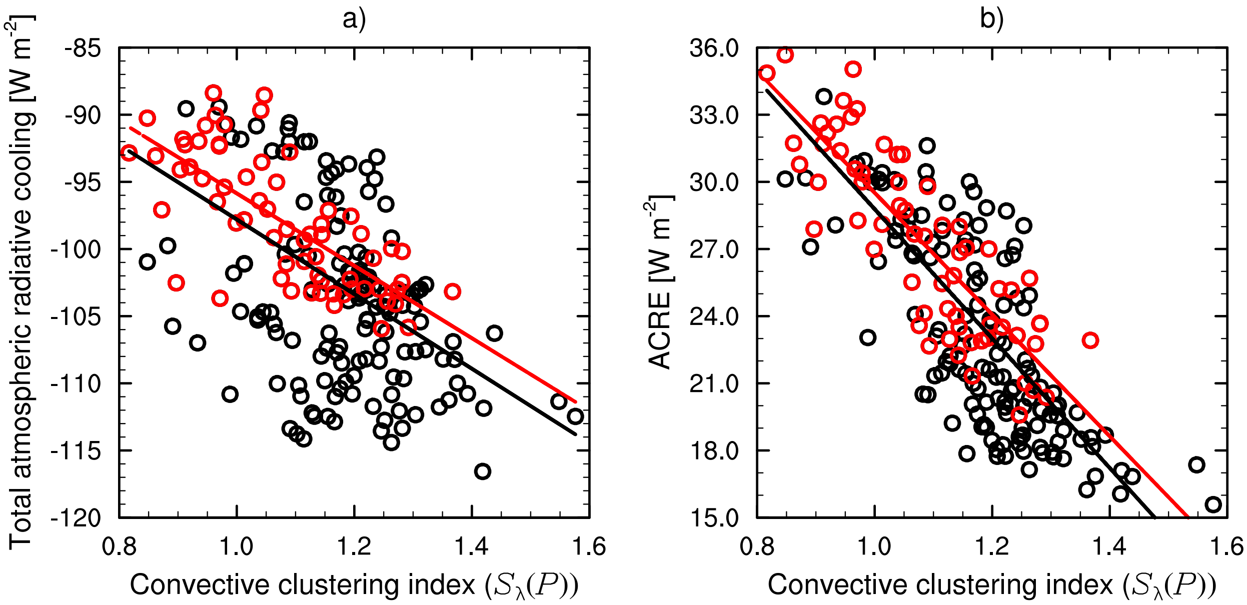

Supplementary Figure 2 | Radiative cooling in observations. Panel a) shows the total vertically integrated atmospheric radiative cooling, panel b) the atmospheric cloud-radiative effect (ACRE) averaged from 6 S to 6 N as a function of the zonal convective clustering ($S_\lambda(P)$) for the GPCP observational dataset from March 2000 to December 2016. The radiative cooling tendencies were calculated from the CERES-EBAF data set. Red circles denote the months with a tropical precipitation distribution that is symmetric about the equator (see methods for details) and black circles those with an asymmetric distribution. The red lines are obtained by linear regression to the red circles and the black lines are obtained by linear regression to all (red and black) circles.

	W_ω	ϕ_S
W_P (GPCP all)	(+) 34%	(+) 4%
W_P (GPCP sym)	(+) 64%	(+) 63%
W_P (Simulations)	(+) 96%	(+) 3%
W_ω (GPCP all)	-	(+) 10%
W_ω (GPCP sym)	-	(+) 52%
W_ω (Simulations)	-	(+) 2%

Supplementary Table 1 | Relationship between the width and the double-peak structure of the intertropical convergence zone. The table shows the variance explained between the precipitation-inferred width of the intertropical convergence zone (ITCZ, W_P), the dynamically inferred width of the ITCZ (W_ω) and the meridional distance between the two zonal-mean peaks in precipitation (ϕ_S). all indicates that the variance explained was calculated using all months, sym using only the months with a precipitation distribution that is symmetric about the equator (see methods) and $Simulations$ using the statistically steady-state mean of the simulations. Note that if there is only one peak in precipitation, the distance between the two peaks is set to zero. The sign indicates if the correlation is positive or negative.

	W_P	W_ω
Amplitude	(+) 50%	(+) 62%
Zonal wavenumber	(-) 23%	(-) 17%

Supplementary Table 2 | Relation between the imposed evaporation forcing width of the intertropical convergence zone The table shows the variance explained between the amplitude as well as the zonal wavenumber of the imposed evaporation forcing in the aquaplanet simulations and the precipitation-inferred (W_P) as well as the dynamically-inferred (W_ω) width of the intertropical convergence zone. The control simulation was excluded from the calculation of the variance explained, because no forcing was imposed. The sign indicates if the correlation is positive or negative.

	Jan	Feb	Mar	Apr	May	Jun	Jul	Aug	Sep	Oct	Nov	Dec
P_{E}	70%	51%	59%	21%	10%	1%	10%	1%	0%	36%	41%	72%
W_P	66%	59%	69%	33%	18%	14%	31%	20%	11%	45%	58%	71%
W_ω	39%	13%	40%	27%	0%	15%	5%	0%	9%	16%	43%	56%

Supplementary Table 3 | Monthly relationship between the zonal convective clustering and the intertropical convergence zone The table shows the variance explained between the zonal convective clustering ($S_\lambda(P)$) in different months and the precipitation at the equator (P_{E}), the precipitation-inferred width of the intertropical convergence zone (ITCZ, W_P) and the dynamically-inferred width of the ITCZ (W_ω).

	$F_{\omega 500>0}$	$F_{0.8\overline{P}}$
$S_\lambda(P)$ (GPCP all)	(+) 73%	(-) 93%
$S_\lambda(P)$ (GPCP sym)	(+) 84%	(-) 96%
$S_\lambda(P)$ (Simulations)	(+) 86%	(-) 92%

Supplementary Table 4 | Relationship between different metrics of zonal convective clustering. The table shows the variance explained between the zonal standard deviation of precipitation normalized by the mean precipitation ($S_\lambda(P)$), the mean area fraction of subsidence ($F_{\omega 500>0}$) and the minimum fraction of surface (and time) necessary to accumulate 80% of the total precipitation ($F_{0.8\overline{P}}$), all calculated in the region from 6 S to 6 N. The sign indicates if the correlation is positive or negative. all indicates that the variance explained was calculated using all months, sym using only the months with a precipitation distribution that is symmetric about the equator (see methods) and $Simulations$ using the statistically steady-state mean of the simulations.
